# Cardiomyopathy induced by premature ventricular contractions with ventricular escape beats in the compensatory pause: A case report and brief review of the literature

**DOI:** 10.1097/MD.0000000000030277

**Published:** 2022-08-26

**Authors:** Yuanjun Sun, Xiaohong Yu, Xianjie Xiao, Shiyu Dai, Rongfeng Zhang, Zhongzhen Wang, Chengming Ma, Xiaomeng Yin, Lianjun Gao, Yanzong Yang, Yunlong Xia

**Affiliations:** a Department of Cardiology, First Affiliated Hospital of Dalian Medical University, Dalian, Liaoning, China.

**Keywords:** ablation, cardiomyopathy, compensatory pause, premature ventricular contraction, ventricular escape beat

## Abstract

We reported a case with cardiomyopathy induced by frequent premature ventricular contractions (PVCs) and followed ventricular escape beats (VEBs). PVCs with VEBs in the compensatory pause which induced cardiomyopathy is rarely reported. Also, the case exhibited many characteristics of PVCs which were more likely to induce cardiomyopathy, like the location of origin, the longer coupling interval, and the QRS wave companied with the P wave.

**Patient concerns:**

A 53-year-old man with left ventricular (LV) dysfunction presented with palpation, chest distress, and dyspnea for 3 years. Holter revealed a high burden of ventricular rhythm of PVCs and another wide QRS patterns (96,562 total beats with 87,330 wide QRS beats in 24 hours). The LV ejection fraction decreased to 34% and the left ventricle, right and left atria all dilated.

**Diagnosis:**

He was diagnosed with PVC-induced cardiomyopathy.

**Interventions:**

The patient experienced intracardiac electrophysiological examination which revealed frequent PVCs followed by VEBs in the compensatory pause. Activation mapping of the PVCS and ablation were performed.

**Outcomes:**

PVCs and VEBs disappeared after ablation. The LV ejection fraction increased to 46% at 2 days after the procedure. The diameters of the right and left atria were also significantly reduced.

**Lessons:**

VEBs may occur during the compensatory pause of PVCs. PVCs with VEBs can lead to a high burden of ventricular rhythm and LV dysfunction. Ablation of the PVCs can also eliminate VEBs and improve the LV function.

## 1. Introduction

A high burden premature ventricular contractions (PVCs) is associated with an increased risk of left ventricular (LV) systolic dysfunction and even results in cardiomyopathy.^[1]^ Reversible LV systolic dysfunction caused by frequent PVCs can be defined as PVC-induced cardiomyopathy. The prevalence of PVC-induced cardiomyopathy in patients with PVC ranges from 4.3% to 33%.^[1–4]^ The high PVC burden is the most common risk factor for LV dysfunction.^[3]^ Ventricular escape beats (VEBs) are usually accompanied by complete atrioventricular block or sinus arrest without junctional escape beats. PVCs with followed VEBs in the compensatory pause which induce cardiomyopathy were rarely reported. Herein, we firstly report a case of cardiomyopathy induced by frequent PVCs followed by VEBs. And the case exhibited many characteristics of PVCs which were more likely to induce cardiomyopathy.

## 2. Case description

**Figure 1.**
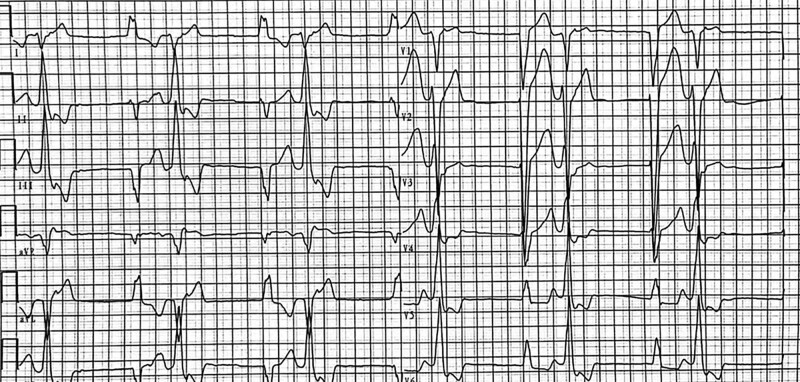
Admission ECG revealed wide QRS of two morphologies without sinus P waves. ECG = electrocardiogram.

A 53-year-old man was admitted for palpation, chest distress, and dyspnea for 3 years with no prior diagnosis and treatment. The symptoms had worsened 2 months prior, accompanied by fatigue without syncope. He did not have any other medical history. He had no family history of cardiomyopathy. Physical examination showed no positive signs except irregular rhythm on chest auscultation. An electrocardiogram (ECG) revealed QRS of two morphologies without sinus P waves (Fig. 1). Transthoracic echocardiography revealed reduced LV ejection fraction (LVEF, 34%) and enlarged LV end-diastolic diameter (58 mm) in sinus rhythm. The left atrial anteroposterior diameter, left-right diameter, and superoinferior diameter were all enlarged (47, 51, and 64 mm, respectively). The right atrial left-right diameter and superoinferior diameter were also enlarged (49 and 61 mm, respectively).

**Figure 2.**
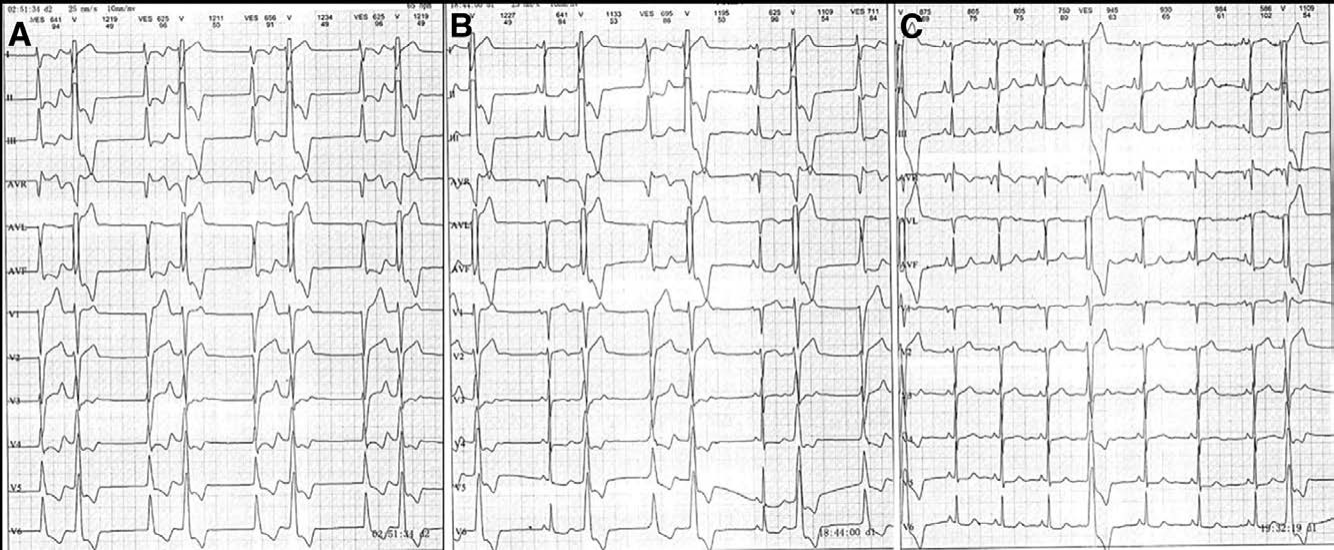
The content of records of the 24-hour Holter. (A) Two wide QRS patterns. (B) PVC with another followed wide QRS pattern in sinus rhythm. (C) Frequent PVCs. PVC = premature ventricular contraction.

Coronary computed tomographic angiography was normal. The patients had no sustained ventricular arrhythmias. The 24 hour Holter revealed a total of 96,562 beats with 87,330 wide QRS beats (Fig. 2).

**Figure 3.**
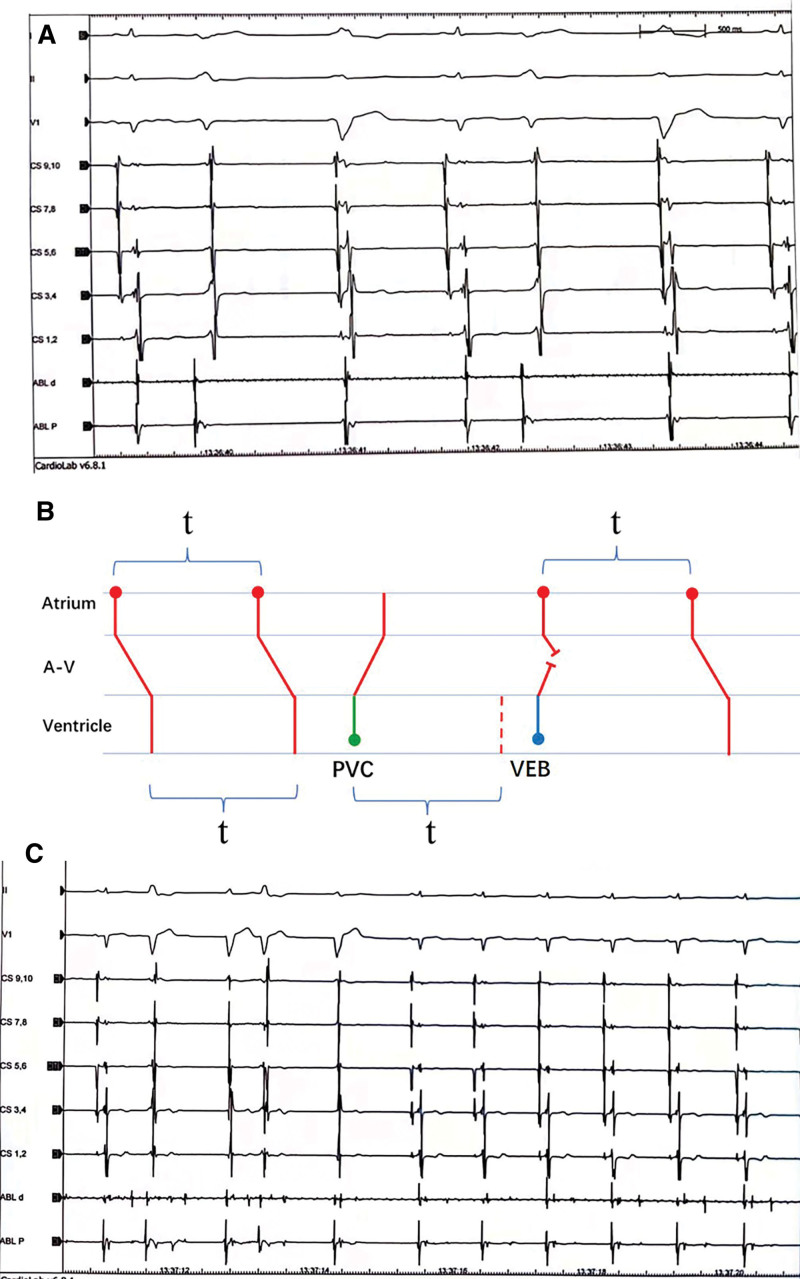
(A) IEGM revealed the two ventricular rhythms. (B) Ladder diagram of mechanism of the two wide QRS patterns. The followed wide QRS pattern was a VEB in the compensatory pause of the PVC. “t” means PP interval in sinus rhythm. No activation conducted to ventricle during the normal “t” and then VEB emerged. (C) The PVCs and the followed VEBs both disappeared after ablation. IEGM = intracardiac electrocardiogram, PVC = premature ventricular contraction, VEB, ventricular escape beat.

No antiarrhythmic drug was used after admission. Intracardiac electrocardiogram revealed the mechanism of the two ventricular rhythms (Fig. 3A). The second wide QRS pattern was VEB in the compensatory pause of the PVC (Fig. 2C) as shown in the ladder diagram (Fig. 3B). Activation mapping of the PVCs was performed by a Thermal-Cool catheter (Biosense Webster, Diamond Bar, CA) guided by the Carto3 system (Biosense Webster, Diamond Bar, CA). The PVCs originated from the septum of the right ventricular outflow tract. PVCs and followed VEBs disappeared after ablation (35–40 W, 48°C) (Fig. 3C). The LVEF increased to 46% at 2 days after the procedure. The diameters of the left atrium (anteroposterior diameter, left-right diameter, superoinferior diameter: 47 mm, 51 mm, 64 mm vs 45 mm, 43 mm, 55 mm) and right atrium (left-right diameter, superoinferior diameter: 49 mm, 61 mm vs 39 mm, 62 mm) all significantly decreased.

## 3. Discussion

A high burden of PVCs independently associated with LV dysfunction.^[3,5]^ The LV function improves after elimination of PVCs. The main reason of PVC-induced cardiomyopathy is LV dyssynchrony. Uncoordinated contractility of LV segments’ results from PVCs is commonly related to the site of origin and coupling interval. PVCs originating from the right ventricle (RV) can cause more severe LV dyssynchrony compared with LV-PVCs in theory. And PVCs originating from the RV are more likely to resulting in LV dysfunction than LV-PVCs.^[6]^ Non-outflow tract origin is also a risk factor of the development of PVC-induced cardiomyopathy.^[7]^ In the present case report, the site of the origin of the PVCs and the VEBs are both located in the RV according to the activation mapping and ECG characteristics. And the VEBs originated in the non-outflow tract of RV because of the negative QRS waves in the inferior wall leads and V1 to V4 leads in the ECG. Besides, late-coupled PVCs demonstrated more obvious LV dyssynchrony and may cause more severe LV dysfunction.^[8]^ Potfay et al^[8]^ found that LV dyssynchrony during PVCs was primarily dependent on the coupling interval. In this case, the coupling interval before the VEBs was significantly prolonged and even longer than the sinus R-R interval, which further promoted LV dyssynchrony. Therefore, the high burden, RV origin of the PVCs and VEBs and the long coupling interval before the VEBs contributed to the LV dysfunction.

According to the intracardiac electrocardiogram and ladder diagram, the VEBs emerged followed by a sinus P wave, which meant the left atrium contracted shortly after or at the same time of the left ventricle contraction. Maybe the left atrium contracted during the mitral valve closing. The phenomenon can cause a pulmonary vein flow reversal and left atrial pressure elevation, which results in left and right atria enlargement.^[9,10]^ After elimination of the PVCs and VEBs, the diameters of the left and right atria significantly reduced.

Normalization of the LVEF commonly occurs within 3 months after elimination of PVCs by ablation.^[11]^ Hasdemir et al^[12]^ reported that more than half of PVC-induced cardiomyopathy patients had ≥25% improvement in LVEF when experienced transthoracic echocardiography at 1 week after ablation. The LVEF increased by 35.3% ([0.46 − 0.34]/0.34) at 2 days after ablation in the present case.

## 4. Conclusion

A high burden of ventricular rhythm from PVCs and VEBs could result in cardiomyopathy in the case report. VEBs disappeared after elimination of PVCs by ablation, and the LV function improved afterward soon.

## Author contributions

**Conceptualization:** Xiaohong Yu, Xiaomeng Yin, Yuanjun Sun.

**Data curation:** Shiyu Dai, Xianjie Xiao.

**Investigation:** Zhongzhen Wang.

**Software:** Rongfeng Zhang.

**Visualization:** Chengming Ma, Yuanjun Sun.

**Writing – original draft:** Yuanjun Sun.

**Writing – review & editing:** Lianjun Gao, Xiaohong Yu, Xiaomeng Yin, Yanzong Yang, Yunlong Xia.
